# Genetic Variation in Pulpwood Properties of Hybrid Larch Families and Their Progenies

**DOI:** 10.3390/plants15020190

**Published:** 2026-01-07

**Authors:** Naizhong Hu, Jiaqi Huang, Guanghao Cao, Panke Yang, Huanzhen Liu, Chunming Li, Wenbo Zhang

**Affiliations:** State Key Laboratory of Tree Genetics and Breeding, Northeast Forestry University, Harbin 150040, China; hu15863387957@163.com (N.H.);

**Keywords:** hybrid larch, families, pulpwood, F_1_ generation, F_2_ generation

## Abstract

This study aimed to elucidate the genetic variation patterns of hybrid larch during generational transitions, providing a theoretical basis for targeted genetic improvement and advanced hybrid breeding of pulpwood. Seven hybrid larch families and their progeny from the Qingshan National Larch Elite Seed Base in Linkou County, Heilongjiang Province, were used as experimental materials. Growth traits, wood properties, and pulping performance of both generations were measured and analyzed, and pulp production capacity was calculated accordingly. Significant differences were observed between the F_1_ and F_2_ generations across all traits. The F_2_ generation showed greater genetic stability, though it was more susceptible to environmental factors. The LK3 × LG2 family was identified as elite through principal component analysis (PCA) and membership function analysis. Further analysis of intergenerational data confirmed that the LK5 × LO78-3 family exhibited superior genetic stability across generations, making it an optimal parental source. This study establishes a genetic foundation for the targeted genetic improvement of larch pulpwood, with important implications for advanced hybrid breeding and elite family selection.

## 1. Introduction

*Larix* spp., a deciduous tree in the Pinaceae family and the genus *Larix*, is a fast-growing species characterized by tall, straight trunks and strong environmental adaptability. It is one of the most widely distributed native tree species in northern China and plays a pivotal role in afforestation efforts, such as farmland-to-forest conversion, shelterbelt establishment, and the creation of fast-growing, high-yield forests [1,2,3]. Furthermore, larch wood is prized for its high cellulose content and ease of processing, making it valuable in the paper, textile, and chemical industries [4,5,6]. As the world’s largest producer and consumer of paper and paperboard, China remains heavily reliant on imported wood pulp, with imports accounting for 54.4% of total consumption in 2023 [7]. Larch, comprising approximately 7.15% of China’s total timber volume, represents a significant resource with immense development potential [8]. Consequently, there is an urgent need to rationally develop and utilize larch timber resources to bridge the domestic wood pulp supply gap.

Due to long generation intervals, extended growth cycles, and complex genomes, studying genetic variation across tree generations remains challenging. However, it is crucial for improving breeding efficiency [9,10,11]. The primary objective of forest tree breeding is to develop superior varieties that meet production requirements in terms of growth, wood quality, and stress resistance, with stable hereditary traits adapted to specific environmental and cultivation conditions. Achieving this requires continuous genetic improvement across generations through iterative breeding cycles, as well as the accumulation and utilization of favorable genetic variations [12]. Research on improving larch varieties and artificial afforestation has progressed since the 1960s, with hybrid breeding studies beginning in the 1980s. Crossbreeding Japanese larch with Changbai and Xing’an larch for the first time harnessed hybrid vigor, producing hybrid families with rapid growth and improved resistance [13]. Extensive studies have confirmed that hybrid larches exhibit significant hybrid vigor in growth rate, stress tolerance, and wood quality [14,15,16,17,18]. Similar advantages have also been observed in other tree species. For instance, in western larch (*Larix* occidentalis), hybrid breeding has produced families with improved drought tolerance and growth performance [19]. Likewise, in South African eucalyptus breeding programs, hybrids of *E. grandis* × *E. camaldulensis* have demonstrated significant enhancements in growth traits, drought resistance, and pulping properties compared to their parental lines [20]. Therefore, interspecific hybridization and genetic improvement of larch are critical. Controlled and open pollination are essential techniques for enhancing seed quality in breeding programs. While controlled pollination efficiently produces desired genotypes and maximizes genetic gain [21], it is labor-intensive, time-consuming, and costly [22]. In contrast, open pollination reduces costs but faces challenges such as pollen contamination.

This study evaluated the growth, wood properties, and pulping characteristics of seven hybrid larch families to further understand the hybrid vigor and genetic variation in hybrid larch under controlled (F_1_ generation) and open pollination (F_2_ generation). The aim was to explore the F_2_ generation’s potential as pulpwood. These findings offer valuable insights into promoting and utilizing the F_2_ generation and provide a theoretical foundation for breeding efforts.

## 2. Results

### 2.1. Comparison of Growth Traits Between F_1_ and F_2_ Generations

Highly significant differences were observed among the seven families in the F_1_ generation and among the 10 families in the F_2_ generation (Table 1). Figure S1 presents a series of comparisons for the growth traits of the F_1_ and F_2_ generations of the seven families under investigation. Regarding tree height, the LK5 × LO77-3 family showed the best performance in the F_1_ generation, while significant variation was observed among the 10 F_2_ families. Among the seven hybrid larch families, tree height was notably greater than in the three control families, with the LK3 × LG2 family demonstrating the most favorable performance. For diameter at breast height, significant differences were found among the seven F_1_ families, with the LK12 × LG9 family exhibiting the best performance, showing a substantial increase compared to most families. In contrast, the LK5 × LO77-3 family performed the poorest, significantly lagging behind the other six families. Among the F_2_ generation, the LK5 × LO77-3 family also demonstrated the poorest performance, significantly lower than the LK3 × LG2 family, although the differences between the LK5 × LO77-3 family and the other five families were not statistically significant. Additionally, all seven hybrid larch families were significantly taller than the three control families. Regarding wood volume, significant differences were observed among the seven F_1_ families. The LK12 × LG9 family outperformed the other six, while the LK5 × LO77-3 family showed the least favorable performance, significantly behind the others. In the F_2_ generation, all seven hybrid larch families performed significantly better than the three control families, but no significant differences were found among the seven hybrid families. The estimated genetic parameters for growth traits in the F_1_ and F_2_ generations of seven families are summarized in Table 1. The PCV for growth traits in the F_2_ generation showed a slight increase compared to the F_1_ generation, although the PCV values for both generations were notably lower than those of the CK. The GCV for growth traits in the F_2_ generation was lower than that in the F_1_ generation, suggesting greater genetic stability in the F_2_ generation. The GCV for wood volume in the CK was significantly lower than in the F_1_ generation. The heritability (*h*^2^) in the F_2_ generation declined compared to both the CK and F_1_ generations. However, it was evident that growth traits in all three generations (F_2_, F_1_, and CK) were subject to substantial genetic regulation.

**Figure 1:**
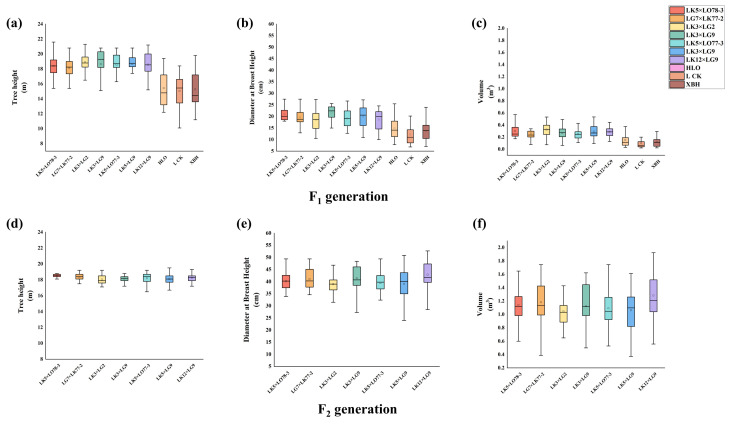
Data Distribution Analysis of Growth Traits in F_1_ and F_2_ Hybrid Larch Families. The figure illustrates the data distribution analysis plots for Tree height (**a**), Diameter at breast height (**b**), and volume (**c**) of the F_2_ generation, as well as tree height (**d**), DBH (**e**), and volume (**f**) of the F_1_ generation in hybrid larch families.

The subsequent analysis of data distribution, presented in Figure 1, revealed that F_1_ data exhibited superior central tendency, dispersion, outlier control, and distribution shape, alongside significantly higher stability compared to the F_2_ data. Nevertheless, among the F_2_ hybrid larch families, data stability was found to be higher in hybrid families than in control families. Specifically, the LK5 × LG9 and LK12 × LG9 families demonstrated high stability across generations, indicating robust genetic stability. In terms of breast height diameter and volume, the F_2_ data outperformed the F_1_ data in central tendency, dispersion, outlier control, and distribution shape. A statistically significant increase in data stability was observed between the F_2_ and F_1_ groups.

### 2.2. Comparison of Wood Property Traits Between F_1_ and F_2_ Generations

Significant differences were observed among the seven F_1_ families and among the ten F_2_ families (Table 2). A comparative analysis of the wood properties in the F_1_ and F_2_ generations of seven families is presented in Figures S2 and S3. Regarding basic density, no substantial differences were found among the seven F_1_ families. In the F_2_ generation, the LG7 × LK77-2 family performed best, though the increase in performance was not significant relative to the other six families. For cellulose content, the LK5 × LO78-3 family exhibited optimal performance among the seven F_1_ families, with a marked increase compared to the other six families. In contrast, the LK5 × LO77-3 family showed the poorest performance, with a significant decrease compared to most families. Among the seven F_2_ families, LG7 × LK77-2 had the highest cellulose content, significantly surpassing most families. The LK5 × LO77-3 family performed the worst, though the difference was not statistically significant compared to several other families. No statistically significant differences were found for hemicellulose content among the seven F_1_ families or between the F_1_ and F_2_ generations. In terms of total cellulose content, the LK5 × LO78-3 family demonstrated the best performance among the seven F_1_ families, although its performance was only significantly higher than that of a few families. The LK3 × LG2 family exhibited the poorest performance, showing a significant decline compared to most families. In the F_2_ generation, the LK12 × LG9 family performed most favorably, significantly outpacing the other six families. Conversely, the LK5 × LO78-3 family showed the least favorable performance, although the deviations from most families were not statistically significant. For lignin content, the LK3 × LG9 family exhibited the best performance among the seven F_1_ families, significantly surpassing most families. In the F_2_ generation, the LK3 × LG2 and LK3 × LG9 families performed better, showing significantly higher results than most of the other families.

Regarding fiber length, the LG7 × LK77-2 family exhibited the best performance among the seven F_1_ families, significantly surpassing most others. In contrast, LK5 × LO78-3 performed the least favorably, falling significantly below the median of the other families. In the F_2_ generation, LK3 × LG2 demonstrated the highest performance, with a marked improvement over the other six families. For fiber width, LK3 × LG2 showed the best performance among the seven F1 families, significantly exceeding several other families. Conversely, LK5 × LO78-3 exhibited the poorest performance, with significant differences observed compared to the majority of families. In the F_2_ generation, the LK3 × LG2 family had a significantly higher fiber width compared to the remaining six families, though no significant differences were found among the latter. Regarding the fiber length-to-width ratio, the differences among the seven families in both the F_1_ and F_2_ generations did not reach statistical significance.

The estimated heritability parameters for wood properties in the F_1_ and F_2_ generations of seven families are presented in Table 2. The PCV and GCV for basic density and fiber components fluctuated by less than 3% between the F_2_ and F_1_ generations, indicating stable inheritance of wood properties across generations. The *h*^2^ for hemicellulose content and total cellulose content was higher in the F_2_ generation than in the F_1_ generation, while the *h*^2^ for lignin content remained consistent between generations. Conversely, the *h*^2^ for cellulose content and basic density was higher in the F_1_ generation than in the F_2_ generation, although both traits showed considerable genetic regulation. The *h*^2^ and stability for hemicellulose and total cellulose content were consistently high across both generations. In contrast, the PCV for fiber length decreased in the F_2_ generation compared to the F_1_ generation, while the GCV and *h*^2^ remained largely stable between generations. The PCV and GCV for fiber width declined in the F_2_ generation compared to the F_1_ generation, with a slight increase in *h*^2^. The PCV, GCV, and *h^2^* for the fiber length-to-width ratio all significantly decreased in the F_2_ generation.

**Figure 2:**
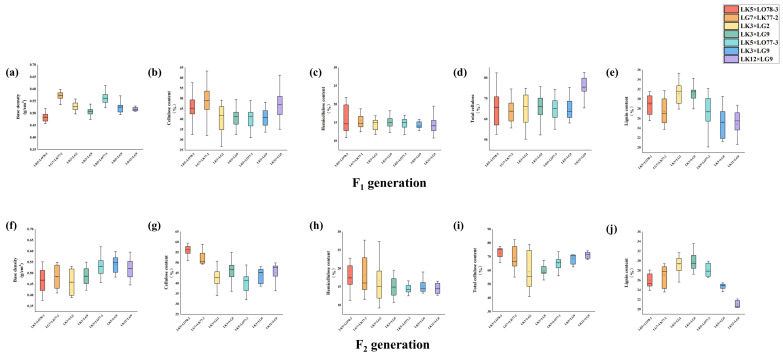
Data Distribution Analysis of Wood Property Traits in F_1_ and F_2_ Hybrid Larch Families. The figure illustrates the data distribution analysis plots for Base density (**a**), Cellulose content (**b**), Hemicellulose content (**c**), Total cellulose content (**d**), and Lignin content (**e**) of the F_2_ generation, as well as Base density (**f**), Cellulose content (**g**), Hemicellulose content (**h**), Total cellulose content (**i**), and Lignin content (**j**) of the F_1_ generation in hybrid larch families.

**Figure 3:**
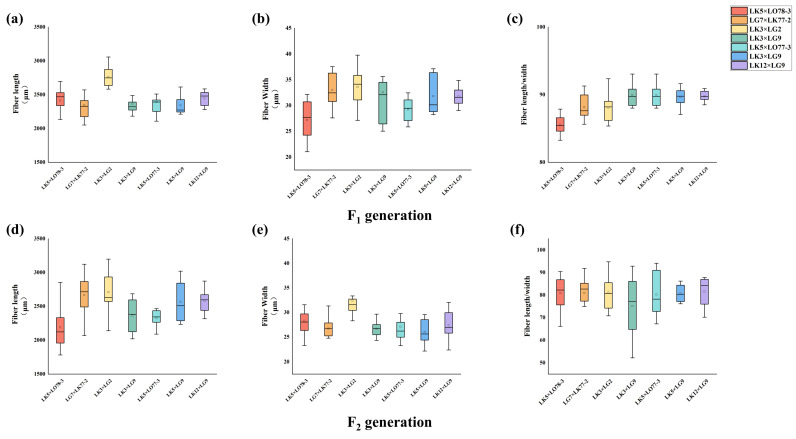
Data Distribution Analysis of Fiber Morphological Characteristics in F_1_ and F_2_ Hybrid Larch Families. The figure illustrates the data distribution analysis plots for Fiber length (**a**), Fiber Width (**b**), and Fiber length/width (**c**) of the F_2_ generation, as well as Fiber length (**d**), Fiber Width (**e**), and Fiber length/width (**f**) of the F_1_ generation in hybrid larch families.

The distribution of wood traits in the F_1_ and F_2_ generations of the seven families is shown in Figure 2 and Figure 3. For basic density and hemicellulose content, the F_2_ data exhibited superior central tendency, dispersion, outlier control, and distribution shape, with an upward trend in data stability. However, for cellulose content, the F_2_ data showed increased dispersion, indicated by a 6.33% higher interquartile range (IQR), reflecting greater data variability. For total cellulose and lignin content, the F_1_ data outperformed the F_2_ data in terms of central tendency, dispersion, outlier control, and distribution shape. The analysis revealed a decline in data stability in the F_2_ generation relative to the F_1_ generation, with the LK3 × LG9 family exhibiting the smallest IQR in both generations. Regarding fiber length and fiber length-to-width ratio, most F_2_ families displayed superior central tendency, dispersion, outlier control, and distribution shape compared to the F_1_ families. The stability indices for the F_2_ generation were higher for most traits, although the F_1_ generation showed greater stability for fiber width. Furthermore, the rankings for fiber width and fiber aspect ratio were nearly identical between the F_1_ and F_2_ generations across most families. However, significant discrepancies in fiber length were observed between the F_2_ and F_1_ generations in the LG7 × LK77-2 family, while other families exhibited consistent patterns.

### 2.3. Comparison of Pulping Performance Traits Between F_1_ and F_2_ Generations

The differences between the seven F_1_ families and the seven F_2_ families were generally not significant (Table 3). The differences observed among the seven F_1_ families were not statistically significant, while the differences among the seven F_2_ families reached significant levels (Table 3).

Figure S4 presents comparisons of the pulping performance traits in the F_1_ and F_2_ generations of the seven families. For crude pulp yield, no significant differences were observed between the F_1_ or F_2_ generations of the families. For fine pulp yield, no significant differences were found among the seven F_1_ families. In the F_2_ generation, the LK12 × LG9 family performed optimally, significantly outperforming the LK5 × LO78-3 family, although differences with other families were not significant. No significant discrepancies were observed in the screen residue rate between the F_1_ and F_2_ generations of the families. Multiple comparisons of pulping performance traits across the seven families in both F_1_ and F_2_ generations are presented in Figure S3. Further analysis of the seven F_1_ families showed no significant differences. In the F_2_ generation, the LK3 × LG2 family exhibited the best performance, with a marked improvement in performance metrics compared to both the LK5 × LO78-3 and LK3 × LG9 families.

The estimated genetic parameters for pulping performance in the F_1_ and F_2_ generations of the seven families are shown in Table 3. The PCV for all traits was higher in the F_2_ generation compared to the F_1_ generation. The GCV for coarse pulp yield and fine pulp yield decreased in the F_2_ generation relative to the F_1_ generation, while the GCV for screen residue rate showed a substantial increase. The *h*^2^ for all traits was higher in the F_2_ generation compared to the F_1_ generation, although the heritability in both generations was relatively low. The fine pulp yield in the F_2_ generation demonstrated higher genetic control, while other traits exhibited moderate to low genetic control. The estimated genetic parameters for pulp productivity traits in the F_1_ and F_2_ generations of seven families are presented in Table 3. The PCV, GCV, and *h*^2^ for pulp yield all showed a significant increase in the F_2_ generation compared to the F_1_ generation.

**Figure 4:**
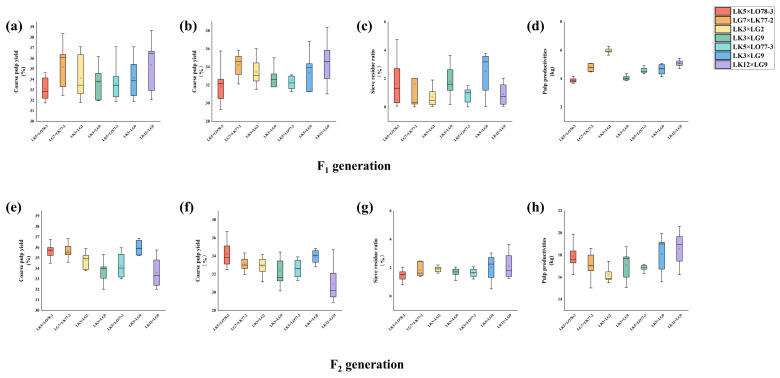
Data Distribution Analysis of Pulping Performance Traits in F_1_ and F_2_ Hybrid Larch Families. The figure illustrates the data distribution analysis plots for Coarse pulp yield (**a**), Fine pulp yield (**b**), Sieve residue ratio (**c**) and Pulp productivities (**d**) of the F_2_ generation, as well as Coarse pulp yield (**e**), Fine pulp yield (**f**), and Sieve residue ratio (**g**) and Pulp productivities (**h**) of the F_1_ generation in hybrid larch families.

Figure 4 presents the distribution analysis of pulping performance traits for the F_1_ and F_2_ generations across the seven families. The F_1_ generation exhibited superior overall performance in terms of central tendency, dispersion, outlier control, and distribution shape for coarse pulp yield, fine pulp yield, and screen residue rate when compared to the F_2_ generation. Data stability in the F_2_ generation showed a declining trend, indicating increased dispersion and trait segregation. However, within the F_2_ generation, the LK3 × LG2 and LK12 × LG9 families demonstrated upward trends in coarse pulp yield, fine pulp yield, and screen residue rate compared to the F_1_ generation. Figure 4 illustrates the distribution of pulp productivity trait data for the F_1_ and F_2_ generations of the seven families. A comparison between the two generations revealed that the F_2_ generation exhibited significantly improved stability in the pulp productivity traits. This was evidenced by an upward shift in median values, narrower data distribution ranges, fewer outliers, and a stronger concentration of high values, demonstrating enhanced genetic stability across families.

### 2.4. Principal Component Analysis of the F_2_ Generation and Comprehensive Evaluation Using the Membership Function Method in the F_2_ Generation

The principal component analysis (PCA) results for 15 traits in the F_2_ generation are presented in Table S1. The four selected principal components had eigenvalues of 5.457, 4.307, 2.871, and 1.805, with contribution rates of 36.379%, 28.710%, 19.137%, and 12.033%, respectively. The cumulative contribution rate reached 96.259%, indicating that these components effectively represented the original 15 measured traits for evaluating hybrid larch.

The comprehensive index values, normalized principal component scores, D-values, and ranking results for each family are presented in Table S2. Based on the PCA results and the membership function comprehensive evaluation formula, the D-values of the seven families were calculated. A higher D-value reflects greater suitability for pulpwood use. The calculated results are shown in Table S2. Among the seven families, LK3 × LG2 exhibited the highest D-value of 0.762, indicating its highest potential for pulpwood utilization in the F_2_ generation.

## 3. Discussion

Wood volume serves as a direct indicator of tree growth and determines pulp yield per unit area; therefore, greater wood volume implies higher potential pulp production [23]. The F_1_ generation exhibited a mean wood volume of 1.12 m^3^, with a phenotypic coefficient of variation (PCV) of 28.13% and Narrow-sense heritability (*h*^2^) of 0.77, indicating superior and stable growth performance, well-defined genetic background, and suitability for propagation as elite clonal material. In contrast, the F_2_ generation showed a higher PCV and lower heritability compared to the F_1_ generation, suggesting that its variation is influenced more by environmental factors than by genetic effects [24]. This may be attributed to the fact that the F_2_ stand, being at a near-mature stage, is more sensitive to environmental conditions than the mature F_1_ stand. Notably, the PCV values of both the F_1_ and F_2_ generations were substantially lower than *olgensis* (CK), indicating relatively lower phenotypic variation and greater stability in growth traits among the hybrid larch families. Furthermore, all growth-related traits in the F_2_ generation exceeded those of CK, confirming the persistence of hybrid vigor in the progeny, which aligns with findings reported by Deng [25] in studies on hybrid larch.

The fiber composition of pulping raw materials significantly influences both pulping processes and pulp properties [26]. Wood primarily consists of cellulose, hemicellulose, and lignin [27]. Cellulose is the main component of pulp, while a higher hemicellulose content can enhance the mechanical strength of paper. In contrast, lignin adversely affects paper strength and tends to oxidize, leading to paper yellowing; therefore, it should be removed as much as possible during pulping [28]. In this study, the F_2_ generation exhibited average cellulose, hemicellulose, and lignin contents of 46.31%, 15.47%, and 28.2%, respectively. The cellulose and hemicellulose contents were relatively high, whereas the lignin content was comparatively low for a coniferous species, indicating that this hybrid larch possesses favorable pulping characteristics [29]. Additionally, a positive correlation was observed between cellulose content and wood volume, consistent with the findings of Deng [25], suggesting the feasibility of simultaneously improving growth traits and wood properties. Wood density plays an important role in pulping efficiency [30]. For modern pulp production, wood with a density below 0.75 g/cm^3^ is generally suitable as pulpwood, with the optimal range typically between 0.4 and 0.6 g/cm^3^ [31]. In this study, the average basic density of hybrid larch was 0.52 g/cm^3^, which is higher than that of Masson pine [32] and poplar [33], implying that less raw wood material is required to produce the same tonnage of pulp. Moreover, the density below 0.6 g/cm^3^ suggests that hybrid larch wood may have the potential to reduce energy and chemical consumption during pulping. The *h*^2^ of basic density in the F_2_ generation was 0.72, higher than that reported by Fukatsu [34] for Japanese larch, indicating a certain degree of hybrid advantage in this trait for hybrid larch. In this study, the average fiber length and width of hybrid larch were 2452.82 μm and 29.02 μm, respectively, aligning with previously reported fiber morphology data for similar species [35]. A higher fiber aspect ratio allows for more frequent fiber intertwining, thereby improving paper strength [36]. The average fiber aspect ratio here was 81.85, exceeding that reported by Wu et al. [37] for poplar. Consequently, pulp produced from hybrid larch exhibits higher tear and tensile strength, making it suitable for paper grades requiring superior mechanical properties. Furthermore, all fiber morphology traits in this study surpassed those reported by Zhang [38] for Changbai larch, suggesting that hybrid larch holds an advantage over Changbai larch in terms of fiber morphology. The phenotypic coefficient of variation (PCV) and genotypic coefficient of variation (GCV) for fiber composition, fiber length, and fiber width varied by less than 3% between the F_2_ and F_1_ generations, indicating stable inheritance of wood property traits across generations. These traits are likely controlled by strong additive gene effects and can serve as reliable indicators for parental selection in advanced-generation breeding programs.

In this study, the coarse pulp yield of hybrid larch ranged from 32.03% to 39.26%, and the fine pulp yield ranged from 30.40% to 37.20%, both lower than the results reported by Shi et al. and Xu et al. [39,40] for Japanese larch. This discrepancy may be due to variations in pulping processes and the older age of the hybrid larch trees. The *h*^2^ of coarse pulp yield ranged from 0.29 to 0.39, and that of fine pulp yield ranged from 0.37 to 0.49, similar to the findings of Raymond et al. and Wu et al. [41,42] for eucalyptus, but higher than those reported by Li [43] for Changbai larch. This suggests that the pulping performance of hybrid larch is less influenced by environmental factors compared to Changbai larch. Notably, most differences in the three pulping performance indicators among families in this study did not reach significant levels, consistent with Li’s findings. Significant differences in pulp yield potential were observed between the F_1_ and F_2_ generations. The PCV, GCV, and *h*^2^ for pulp yield potential increased in the F_2_ generation compared to the F_1_, with higher data stability observed in the F_2_ generation. This trend was consistent across all families. In this study, pulp yield potential ranged from 13.03 to 20.59 in the F_1_ generation, while in the F_2_ generation, it ranged from 3.67 to 6.28. The pulp yield potential reported by Luo [44] for eucalyptus clones ranged from 22.40 to 46.74, which is higher than the values observed in this study. However, the variation in pulp yield potential among families in this study was smaller.

Pulp production (PP) integrates wood volume, wood density, and pulp yield, serving as a composite indicator that reflects the pulp output potential per unit forest area. As such, it better represents the practical economic return of breeding efforts. Compared to the F_1_ generation, the F_2_ generation exhibited an increasing trend in PCV, GCV, and *h*^2^ for PP. Data stability was also higher in the F_2_ generation, and this pattern was consistent across families. In this study, PP ranged from 13.03 to 20.59 in the F_1_ generation and from 3.67 to 6.28 in the F_2_ generation. While these values are lower than those reported by Luo [44] for eucalyptus clones (22.40–46.74), the PP in the present study showed less variation among families, indicating that selection could lead to the development of elite varieties with more uniform traits and stable output.

Notably, the LK5 × LO78-3 family exhibited low data dispersion and ranked among the top-performing families in both growth and wood property traits across the F_1_ and F_2_ generations. It also performed well in the comprehensive evaluation using the membership function method in the F_2_ generation. These results indicate that this family maintained relatively stable genetic performance across generations, suggesting its potential as ideal material for preserving and disseminating superior genotypes [45].

Unlike prior studies, this research focuses on hybrid larch families and their progeny, providing a comprehensive analysis of multiple traits directly related to pulpwood production, including growth, wood properties, pulping performance, and pulp productivity. The scope of this study is more systematic and thorough compared to earlier investigations.

## 4. Materials and Methods

### 4.1. Plant Materials and Study Site

Plant materials were sourced from full-sib and half-sib family trial forests within the Qingshan National Larch Seed Orchard, located in Linkou County, Heilongjiang Province (129°33′ E, 44°39′ N). The region, situated at an elevation of 400 m, experiences an annual mean temperature of 2.6 °C, annual precipitation of 500 mm, an accumulated temperature of 2390 °C (with a threshold of 10 °C), and a frost-free period of 110–120 days.

The experimental materials included 44-year-old F_1_ progeny from seven hybrid larch families, established in a controlled-pollination plantation. The trial followed a completely randomized block design with four replications. Each plot consisted of three rows, totaling 90 trees, spaced 1.5 × 2.0 m apart. Additionally, the trial incorporated a 31-year-old F_2_ generation from an open-pollinated plantation, also designed as a completely randomized block with five replications and 20-tree plots, with similar spacing of 1.5 × 2.0 m between trees. Both the F_1_ and F_2_ generations were from mature or near-mature plantations [46], with family codes outlined in Table 4 The F_2_ generation controls included HLO, L CK, and XBH.

Under consistent site conditions, ten healthy and well-established individuals per family were selected for growth trait measurements. For both hybrid larch F_1_ and F_2_ generations, six first-order lateral branches (approximately 2 cm in diameter) were collected from the base of each tree to assess wood properties and pulp production performance. Plant material was sampled in late April 2024.

### 4.2. Materials

#### 4.2.1. Measurement of Growth Traits

A comprehensive forest survey of tree height and diameter at breast height was conducted on the F_1_ and F_2_ generations of seven larch families at the Qingshan National Larch Seed Orchard in Linkou County. Timber volume was calculated using the formula: volume = (H + 3)g1.3f, where g1.3 represents the cross-sectional area at breast height and f is the average trial form factor for larch (0.41).

#### 4.2.2. Measurement of Wood Property Traits

Basic density was determined according to the national standard (GB/T 1993-2011) [47]. Cellulose content, hemicellulose content, total cellulose content, and lignin content were measured using the ANKOW 2000i (ANKOM Technology (China), Beijing) fully automatic fiber analyzer. Fiber length and width were assessed with a ZEISS stereomicroscope, measuring 30 complete fibers per sample. The fiber length-to-width ratio was calculated as: Fiber length-to-width ratio = fiber length ÷ fiber width. All measurements were performed in triplicate for each parameter and sample.

#### 4.2.3. Measurement of Pulping Performance Traits

The cooking process employed the sulfate method, with an alkali charge of 18% (as Na_2_O), a sulfation degree of 25%, a liquid-to-solid ratio of 3.5:1, a maximum temperature of 170 °C, natural heating, and a retention time of 120 min. This method utilized T01-15-type digesters and TD3-A-type small digester clusters. Solid residue was analyzed to determine the yield of crude and thin pulp and the screen residue rate. Each indicator was tested with a single replicate per sample.

#### 4.2.4. Calculation of Pulp Productivity Trait

Pulp productivity = Volume × Determine the basic density × Fine pulp yield [44].

#### 4.2.5. Data Analysis

Data were organized using Excel and analyzed for variance using SPSS 27.0. To meet the homogeneity of variance assumption for percentage data, all percentage values were subjected to arcsine square root transformation prior to ANOVA. Differences among families were assessed for significance using Duncan’s multiple range test at α = 0.05. Bar charts and box plots were generated using Origin 2024.

Narrow-sense heritability:

$$h^{2\;}\;=\;1-1/F$$

F—F-value.

PCV (Phenotypic Coefficient of Variation), GCV (Genetic Coefficient of Variation) [48]:

$$\mathrm{PCV}\;=\;\sqrt{\sigma_{p}^{2}}/\bar{X}\times 100\%$$

$$\mathrm{GCV}=\sqrt{\sigma_{f}^{2}}/\bar{X}\times 100\%$$

$\sigma_{f}^{2}$—family variance; $\sigma_{p}^{2}$—phenotypic variance; $\bar{X}$—mean value of the trait for the population.

Membership Function Analysis, Weight Calculation Formula, Comprehensive Evaluation [49]:

$$U\left(\mathrm{Xj}\right)\;=\;\frac{\mathrm{Xj}-\mathrm{Xjmin}}{\mathrm{Xjmax}-\mathrm{Xjmin}}$$

$$\mathrm{Wj}=\mathrm{Pj}/\sum_{i=1}^{n}P$$

$$D=\sum_{i=1}^{n}[U(\mathrm{Xj})\cdot w_{j}]$$

Xj—the j-th comprehensive index; Xj min—the minimum value of the j-th comprehensive index; Xj max—the maximum value of the j-th comprehensive index; Xj—the weight of the j-th comprehensive index among all indices; Xj—the contribution rate of the j-th comprehensive index for each family; Xj—the comprehensive evaluation value.

## 5. Conclusions

This study examined genetic variation in pulpwood-related traits among hybrid larch families and their progeny. The main conclusions are as follows:

The F_2_ generation exhibited greater genetic stability across most traits, as reflected in its generally lower genotypic coefficient of variation (GCV) compared to the F_1_ generation, while maintaining relatively high heritability. This suggests that traits in the F_2_ generation are strongly governed by genetic control, favoring the stable transmission of favorable characteristics.

Based on principal component analysis and comprehensive evaluation using the membership function method, the LK3 × LG2 family was identified as the top-performing pulpwood family in the F_2_ generation. It demonstrated outstanding integrated performance in growth, wood properties, and pulping traits, exhibiting strong potential as an elite cultivar for high-yield, high-quality pulp production. Furthermore, from the perspective of intergenerational genetic stability, the LK5 × LO78-3 family displayed consistent and stable trait performance with minimal genetic variation between the F_1_ and F_2_ generations, making it an ideal candidate for advanced-generation hybrid breeding and parental selection.

This study has established a significant genetic foundation for the targeted breeding of larch pulpwood. It elucidated the selective value of the F_2_ generation in improving pulpwood-related traits and provided theoretical support for parental selection and the formulation of early-selection strategies in advanced-generation breeding programs.

Future research will include additional hybrid larch families with broader genetic backgrounds, expand study areas to systematically investigate the adaptability of target species under varying ecological conditions, and implement long-term monitoring of the tested families and their progeny. This approach will enable a comprehensive and accurate evaluation of the genetic stability of hybrid larch.

## Figures and Tables

**Table 1:** ANOVA of Growth Traits in Hybrid Larch F_1_/F_2_ Families and Gauging of the Heritability Parameters of Growth Traits.

Index	F_2_						F_1_						CK		
	MS	F	P	PCV	GCV	*h* ^2^	MS	F	P	PCV	GCV	*h* ^2^	PCV	GCV	*h* ^2^
H	134.894	43.995	<0.001	8.20%	0.09%	0.57	2.258	3.969	0.001	4.29%	0.26%	0.75	15.44%	2.04%	0.72
DBH	433.903	30.236	<0.001	18.23%	2.62%	0.67	108.901	3.707	0.001	13.92%	5.50%	0.73	32.29%	9.08%	0.74
V	0.283	24.198	<0.001	39.71%	0.10%	0.54	0.403	4.354	<0.001	28.13%	3.56%	0.77	77.69%	0.36%	0.67

MS: Mean Square, PCV: Phenotypic Coefficient of Variation, GCV: Genetic Coefficient of Variation, *h*^2^: Narrow-sense heritability, H: Tree height, DBH: Diameter at Breast Height, V: Volume.

**Table 2:** ANOVA of Wood Property Traits in Hybrid Larch F_1_/F_2_ Families and Gauging of the Heritability Parameters of Wood Property Traits.

Index	F_2_						F_1_					
	MS	F	P	PCV	GCV	*h* ^2^	MS	F	P	PCV	GCV	*h* ^2^
BD	0.012	3.52	0.004	11.60%	0.14%	0.72	0.003	1.134	0.387	11.32%	0.71%	0.12
CC	0.012	3.388	0.003	13.08%	0.08%	0.70	0.029	7.092	<0.001	10.73%	0.47%	0.86
HCC	0.14	52.312	0.877	7.47%	2.52%	0.24	0.084	12.989	0.728	11.02%	2.16%	0.23
TCC	0.321	31.38	<0.001	7.47%	2.01%	0.97	0.261	12.95	<0.001	7.11%	3.88%	0.92
LC	0.01	11.566	<0.001	13.18%	0.23%	0.91	0.007	11.529	<0.001	12.34%	0.22%	0.91
FL	267,100.242	4.806	<0.001	10.85%	36.96%	0.79	267,100.242	5.988	<0.001	13.29%	37.46%	0.83
FW	40.768	5.061	<0.001	11.61%	9.26%	0.80	40.768	4.415	0.001	13.45%	19.10%	0.77
FL/W	35.983	1.346	0.246	5.91%	0.82%	0.26	35.983	2.237	0.062	9.00%	6.18%	0.67

MS: Mean Square, PCV: Phenotypic Coefficient of Variation, GCV: Genetic Coefficient of Variation, *h*^2^: Narrow-sense heritability, BD: Base density, CC: Cellulose content, HCC: Hemicellulose content, TCC: Total cellulose content, LC: Lignin content, FL: Fiber length, FW: Fiber width, FL/W: Fiber length/width.

**Table 3:** ANOVA of Pulping Performance Traits in Hybrid Larch F_1_/F_2_ Families and Gauging of the Heritability Parameters of Pulping Performance Traits.

ANOVA Index	F_2_						F_1_					
	MS	F	P	PCV	GCV	*h* ^2^	MS	F	P	PCV	GCV	*h* ^2^
CPY	0.01	1.641	0.098	6.36%	0.45%	0.39	3.773	1.408	0.009	3.59%	1.80%	0.29
FPY	0.01	1.966	0.04	6.23%	0.47%	0.49	2.512	1.588	0.194	4.03%	0.64%	0.37
SRR	0.001	1.45	0.221	14.30%	1.39%	0.31	0.489	1.127	0.377	7.04%	1.87%	0.11
PP	2.379	32.447	<0.001	14.70%	9.78%	0.97	3.366	1.806	0.134	8.39%	1.73%	0.45

MS: Mean Square, PCV: Phenotypic Coefficient of Variation, GCV: Genetic Coefficient of Variation, *h*^2^: Narrow-sense heritability, CPY: Coarse pulp yield, FPY: Fine pulp yield, SRR: Sieve residue ratio, PP: Pulp production.

**Table 4:** Cross-Reference of Family and Abbreviations.

Family	Abbreviations
*L. kaempferi* 5 × *L. olgensis* 78-3	LK5 × LO78-3
*L. gmelinii* 7 × *L. kaempferi* 77-2	LG7 × LK77-2
*L. kaempferi* 3 × *L. gmelinii* 2	LK3 × LG2
*L. kaempferi* 3 × *L. gmelinii* 9	LK3 × LG9
*L. kaempferi* 5 × *L. olgensis* 77-3	LK5 × LO77-3
*L. kaempferi* 5 × *L. gmelinii* 9	LK5 × LG9
*L. kaempferi* 12 × *L. gmelinii* 9	LK12 × LG9
Hybrid *L. olgensis* orchard seed	HLO
*olgensis control*	L CK
Xiaobeihu *L. olgensis* provenance	XBH

## Data Availability

All data supporting the findings of this study are available within the paper and Supplementary Materials.

## Supplementary Materials

The following supporting information can be downloaded at https://www.mdpi.com/article/10.3390/plants15020190/s1, Figure S1: Multiple comparison of growth traits in F_1_ and F_2_ generations; Figure S2: Multiple comparison of fiber morphological characteristics in F_1_ and F_2_ generations; Figure S3: Multiple comparison of pulping performance traits in F_1_ and F_2_ generations; Figure S4: Multiple Comparison Plot of Pulping Performance Traits; Table S1: Principal component analysis of different families in the F_2_ generation; Table S2: Composite index values, normalized principal component scores, D values, and ranking of families in the F_2_ generation.
