# Supplementary material for: Genetic Variation in Pulpwood Properties of Hybrid Larch Families and Their Progenies
Source: Plants (Basel). 2026 Jan 7;15(2):190. doi: 10.3390/plants15020190 (PMC12844769; doi:10.3390/plants15020190)
Supplement: Supplementary file 1 [file plants-15-00190-s001.zip › Supplementary Figures.pdf]

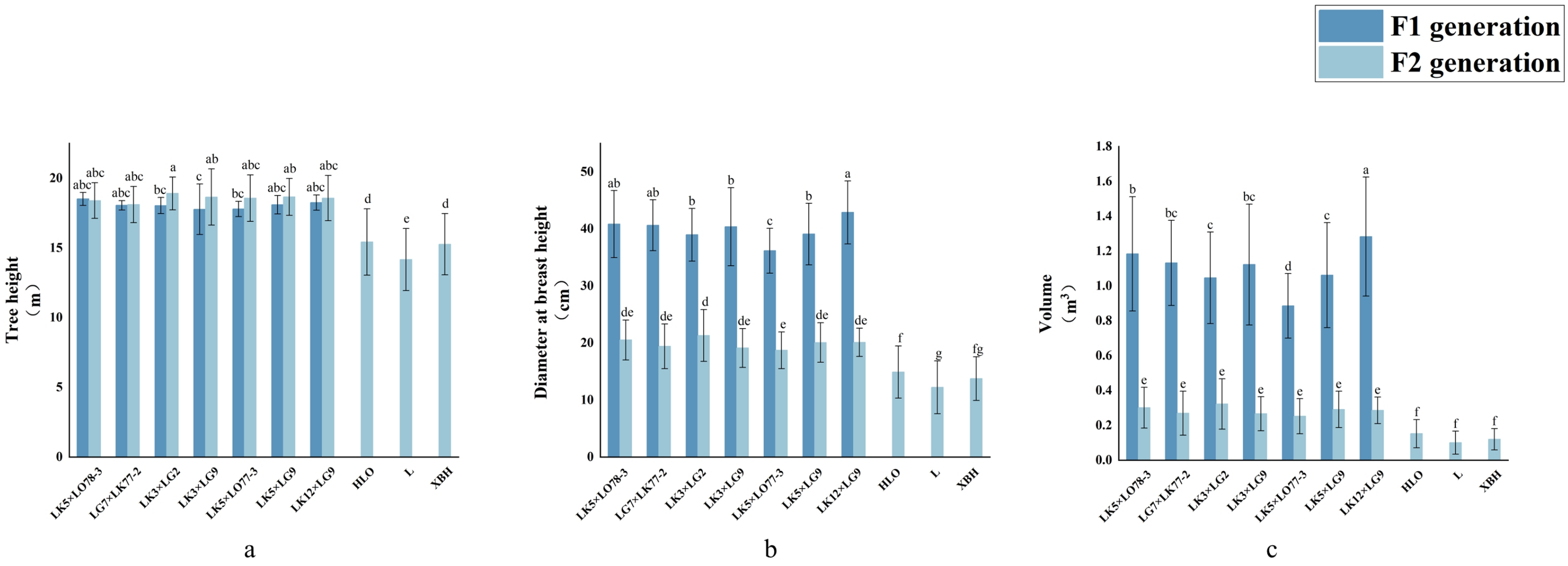

Fig.S1 Multiple Comparison Plot of Growth Traits. Comparison of Tree height (a), Diameter at breast height (b), and Volume (c) among families of F_1 and F_2 hybrid larch

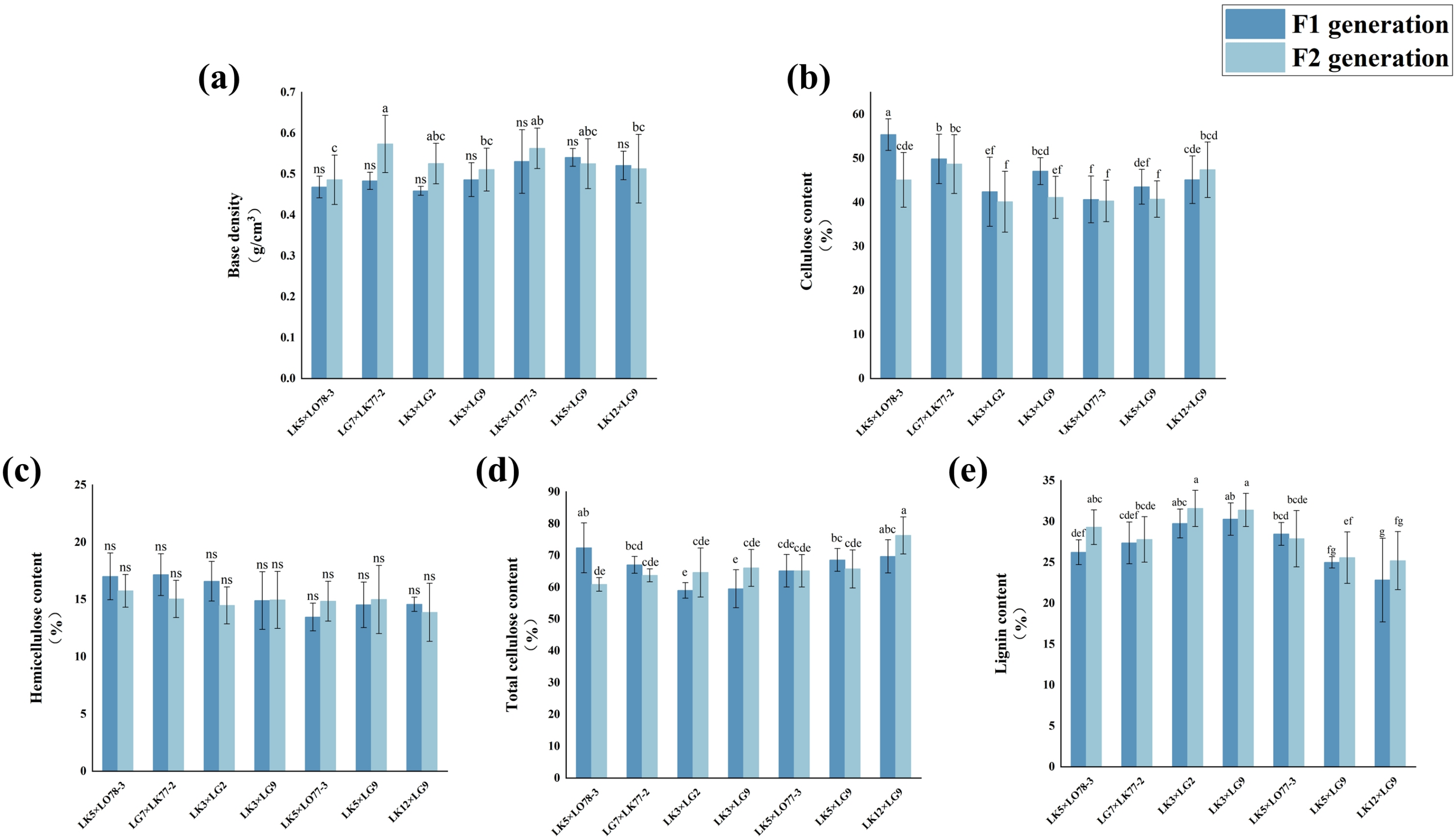

Fig.S2 Multiple Comparison Plot of Wood Property Traits. Comparison of Cellulose (a), Hemicellulose (b), Total cellulose (c), Base density (d), and Lignin (e) among families of F_1 and F_2 hybrid larch

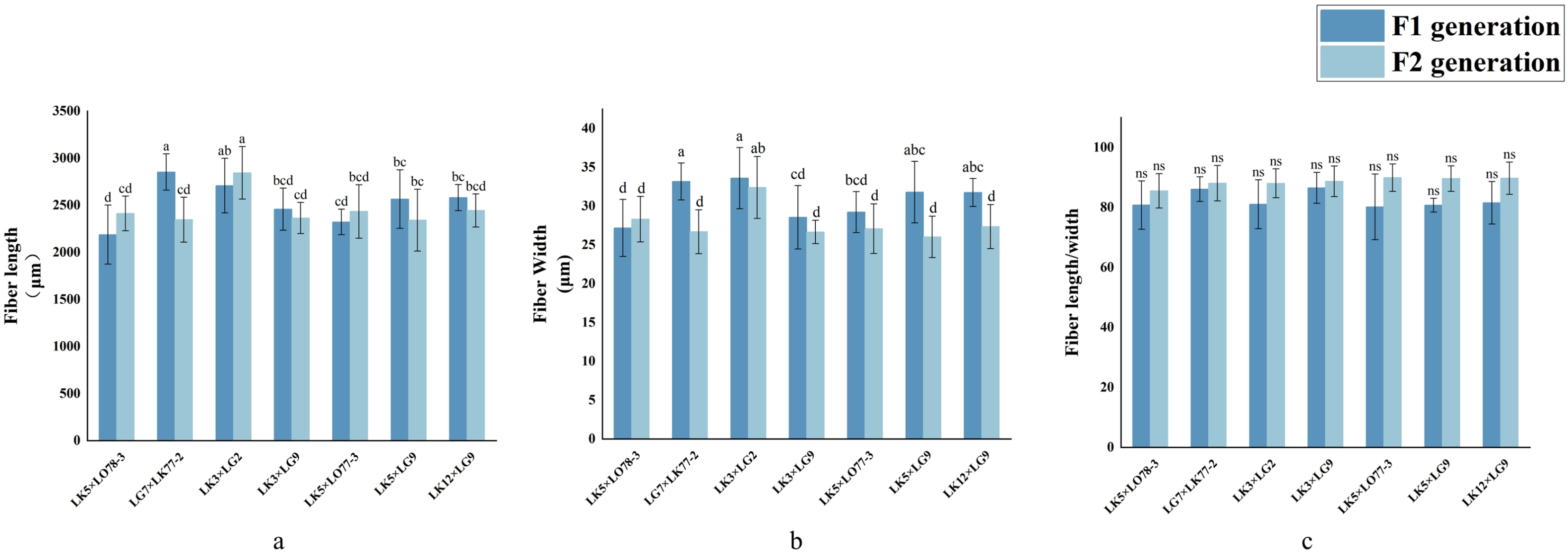

Fig.S3 Multiple Comparison Plot of Fiber Morphological Characteristics. Comparison of Fiber length (a), Fiber Width (b), and Fiber length/width (c) among families of F_1 and F_2 hybrid larch

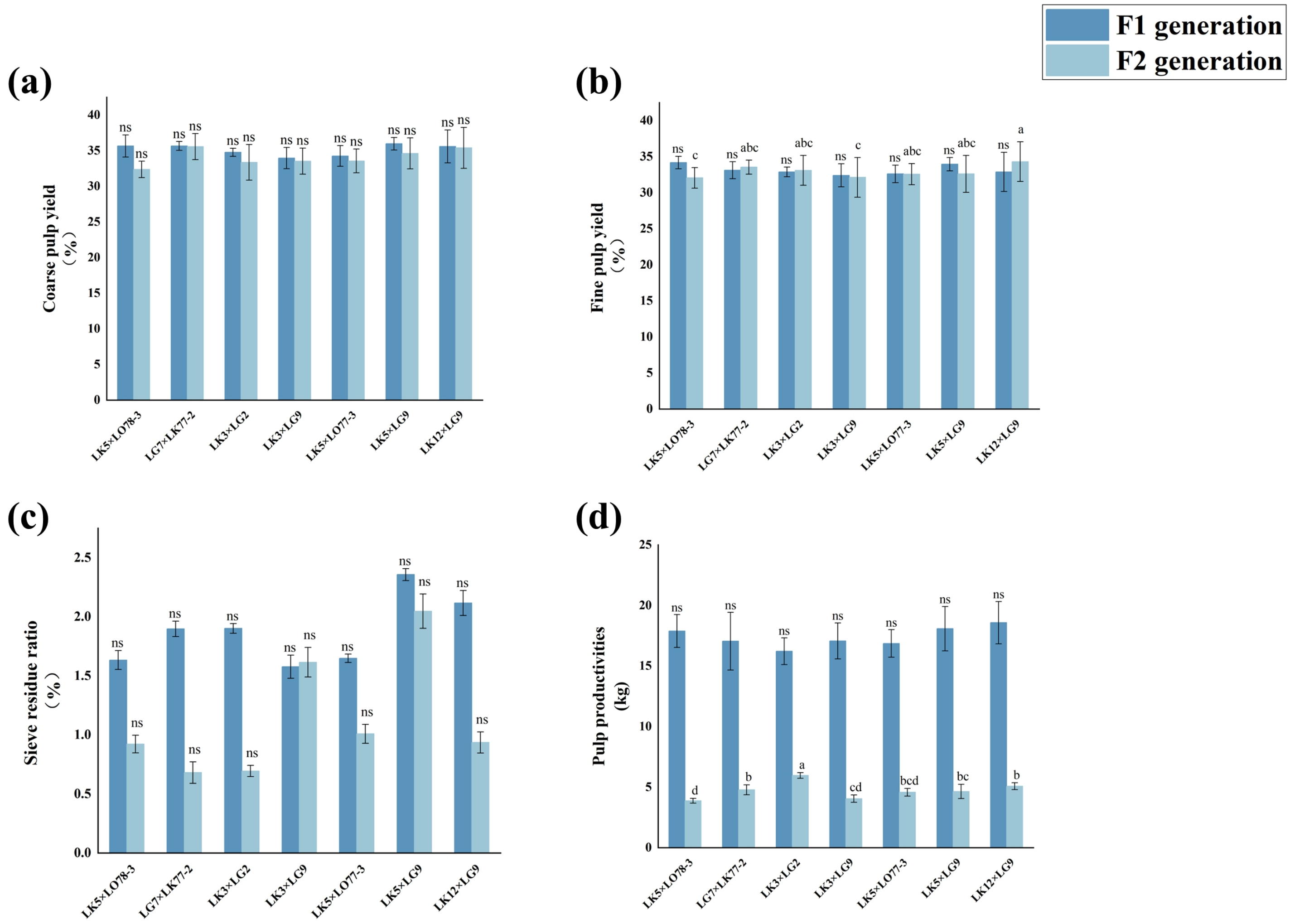

Fig.S4 Multiple Comparison Plot of Pulping Performance Traits. Comparison of Coarse pulp yield(a), Fine pulp yield (b), Sieve residue ratio (c) and Pulp productivities (d) among families of F_1 and F_2 hybrid larch
